# Capsular Switching in Invasive *Neisseria meningitidis*, Brazil^1^

**DOI:** 10.3201/eid1808.111344

**Published:** 2012-08

**Authors:** Terezinha M. P. P. Castiñeiras, David E. Barroso, Jane W. Marsh, Mary M. Tulenko, Mary G. Krauland, Maria C. Rebelo, Lee H. Harrison

**Affiliations:** Federal University of Rio de Janeiro School of Medicine, Rio de Janeiro, Brazil (T.M.P.P. Castiñeiras);; Oswaldo Cruz Institute, Rio de Janeiro (D.E. Barroso);; University of Pittsburgh, Pittsburgh, Pennsylvania, USA (J.W. Marsh, M.M. Tulenko, M.G. Krauland, L.H. Harrison);; and Central Laboratory Noel Nutels, Rio de Janeiro (M.C. Rebelo)

**Keywords:** Meningococcal infections, Neisseria meningitidis, capsular switching, serogroup C, ST-32/ET-5 complex, bacteria, Brazil

## Abstract

During the 1990s, an epidemic of B:4 *Neisseria meningitidis* infections affected Brazil. Subsequent increase in C:4 disease suggested B→C capsular switching. This study identified B→C switches within the sequence type 32 complex. Substantial disease related to capsular switching emphasizes the need for surveillance of circulating meningococcal strains to optimize disease control.

The species *Neisseria* *meningitidis* includes successful commensal strains and devastating human pathogens (*1**–**3*). Invasive strains produce a polysaccharide capsule, which is essential for virulence (*3*) and is a target for most licensed vaccines. Characteristic genomic fluidity (e.g., facilitated by transformation, slipped-strand mispairing, or 2-component regulatory pathways) enables *N. meningitidis* to alter its antigenic profile (*1*). Capsular switching, which occurs through transformation and horizontal gene transfer, enables *N. meningitidis* to escape from host defenses against the original serogroup. This event is common in the absence of vaccination and has implications for meningococcal vaccines that do not cover all serogroups (*4**–**6*).

Brazil experienced a prolonged epidemic of B:4 *N. meningitidis* infections during 1988–1999. In 1990 and 1994, mass vaccination was performed by using a vaccine consisting of a serogroup B outer membrane vesicle (B:4:P1.15) and a serogroup C polysaccharide. During 1993–1994, a total of 4 C:4 *N. meningitidis* isolates were identified in samples from patients in Rio de Janeiro State (RJ). The presence of class3-PorB in these strains suggested the possibility of B→C capsular switching (*7*). Since 2000, the number of cases of serogroup C disease has steadily increased in RJ (*8*), reaching 90% of laboratory-confirmed cases in 2009. Concomitantly, the proportion of C:4 isolates increased from 2% (1988–1999) to 25% (2000–2009). To determine whether C:4 strains arose as a result of B:4 capsular switching, we performed molecular characterization of these isolates.

## The Study

The study was performed at Oswaldo Cruz Institute, RJ, Brazil. The case definition for invasive meningococcal disease was isolation of *N. meningitidis* from a normally sterile body fluid from an RJ resident. Patient characteristics were obtained from the epidemiologic records of the RJ Department of Health during 1988–2009 and analyzed with EpiInfo (version 3.5.3; Centers for Disease Control and Prevention, Atlanta, GA, USA). This study was approved by the Ethical Committee of the Evandro Chagas Research Institute of the Oswaldo Cruz Foundation (protocol 0070.0.009.000–10).

Meningococcal isolates were serogrouped by agglutination and serotyped and serosubtyped by dot blot. The C:4 phenotype was used as a marker of isolates that could have resulted from B:4 capsular switching. Of 41 C:4 isolates recovered from RJ patients during the study period, 35 were viable for further characterization. We also randomly selected 35 B:4 isolates (≈10%) from a list of B:4 isolates available in the laboratory collection from the same period by using SPSS-15 for Windows (SPSS Inc., Chicago, IL, USA).

Multilocus sequence typing (MLST) (*9*) was performed in combination with outer membrane protein (OMP) gene sequencing (*10*) of *porA* variable regions (VRs) 1 and 2, *porB*, and *fetA* VR. Serogroup-specific PCR (*8*) and MLST were repeated for isolates with a suspected capsular switch by using the same template DNA. The assignment to allele, sequence type (ST), clonal complex (CC), *porA, porB,* and *fetA* was performed by querying the Neisseria Sequence Typing Home Page (http://pubmlst.org/neisseria).

STs were considered part of the same clonal complex if they shared at least 4 alleles of the 7 MLST loci with the designated central genotype. OMP gene sequence results were expressed as *porB*:P1, *porA-*VR1, *porA-*VR2, and F.*fetA-*VR (*10*). Meningococcal capsular switching was presumed to have occurred when an ST within a serogroup not usually associated with that ST and more frequently associated with another serogroup was found. MLST and OMP genotypes were used to define capsular switching in specific clones.

The genotyping profiles of 35 C:4 isolates (1993–2009) were the following: C:4.7:P1.7.1 (54%), C:4.7:P1.19.15 (21%), C:4.7:nt (11%), C:4.7:P1.9 (5%), and single genotypes (9%): C:4.7:P1.3,. C:4.7:P1.5, and C:4.7: P1.16. Profiles of the 35 B:4 isolates collected during1988–2009 were: B:4.7:P1.19.15 (48%), B:4.7:P1.7.1 (26%), B:4.7:nt (17%), and single genotypes (9%) B:4.7:P1.7.13, B:4.21:P1.15, and B:4.23:P1.19. A comparison of patients infected by C:4 (n = 30) versus B:4 (n=179) strains showed that the former were more likely to be 5–14 years of age (Table 1). Sex distribution, case-fatality rates, and clinical features were similar for the 2 groups.

**Table 1:** Characteristics of patients with confirmed serogroup B:4 and serogroup C:4 meningococcal disease, Rio de Janeiro, Brazil, 2000–2009

Characteristic	No. (%) serogroup B:4, n = 179	No. (%) serogroup C:4, n = 30	p value
Female sex	93 (52.0)	20(66.7)	0.14
Age group, y			0.046*
<1	27 (15.1)	1 (3.3)	
1–4	50 (27.9)	7 (23.3)	
5–14	56 (31.3)	15(50.0)	
15–24	24 (13.4)	4 (13.4)	
25–64	22 (12.3)	3 (10.0)	
Deceased	30 (16.8)	7(23.3)	0.38
Clinical features			0.11†
Septicemia without meningitis	22 (12.3)	7(23.3)	
Meningitis + septicemia	93 (51.9)	12(40.0)	
Meningitis only	64 (35.8)	11(36.7)	

*p value for comparison of 5–14 y versus all other age groups combined. †p value for comparison of septicemia without meningitis versus other clinical syndromes combined.

Two case clusters of C:4.7:P1.7.1 meningococcal disease occurred in RJ. The first cluster occurred during 2006 at 1 workplace and involved 4 young women. All survived; 1 was left with neurologic deficits. The second cluster occurred in 2009 in a neighborhood and involved 6 children 2–12 years of age and 1 adult. Three patients died.

MLST results showed that of the 35 B:4 isolates, 30 (86%) belonged to ST32CC and most were ST33 and ST639. There were 2 predominant OMP profiles: 3–1: P1.19.15: F5–1 (47%) and 3–79: P1:7–1.1: F5–1(33%), which are consistent with Brazil B epidemic clones. The other STs were single locus variants of ST32 or ST33. Of the 5 remaining isolates, 3 belonged to ST41/44CC, 1 to ST35CC, and 1 to ST3766 (no defined CC).

For C:4 isolates, 29 (83%) of the 35 belonged to the ST32CC. These isolates were detected first in 1993 and became common from 2000 onward. The ST32CC was represented by 6 STs; STs 639 and 33 (79%) predominated. The 2 most common OMP profiles were 3–1: P1.19.15: F5–1 and 3–79: P1:7–1,1: F5–1 (79%). Other STs detected in the ST32CC were ST34 (1 isolate) and 3 new STs (5 isolates): ST7692, ST7696, and ST7709. Of the 6 remaining isolates, 1 belonged to ST41/44CC (ST41) and 5 did not belong to a defined CC: ST7690 (3 isolates), ST7712 (1 isolate), and ST7691 (1 isolate).

OMP genotyping data demonstrated capsular switching in 4 specific meningococcal clones (Table 2). The switch to the ST639 (32CC), 3–79:P1.7–1.1:F5–1 clone resulted in a substantial number of cases and was the cause of the 2009 cluster. A highly related clone (3–79:P1.7–1.1:F5–1:ST-7696), which is a single-locus variant by MLST of the 2009 outbreak clone, was responsible for the 2006 cluster.

**Table 2:** Serogroup B→C capsular switching among *Neisseria meningitidis* isolates belonging to 4 ST32 clonal complex populations with identical OMP genotypes, Rio de Janeiro, Brazil, 1988–2009*

ST and OMP genotype profile	No. isolates	Year(s) of isolation
ST33, 3–1:P1.19,15:F5–1		
Serogroup B:4	13	1988–2009
Serogroup C:4	2	2004–2005
ST34, 3–1:P1.19,15:F5–1		
Serogroup B.4	3	2008–2009
Serogroup C.4	1	2005
ST639, 3–79:P1.7–1,1:F5–1		
Serogroup B:4	10	1989–1997
Serogroup C:4	13	1998–2009
ST639, 3–294:P1.7–1,1:F5–1		
Serogroup B:4	1	1994
Serogroup C:4	1	2008

*ST, sequence type; OMP, outer membrane protein.

## Conclusions

This study demonstrated that after a prolonged epidemic of serogroup B ST32 complex *N. meningitidis* in Brazil, isolates genetically indistinguishable, but expressing the serogroup C capsule, emerged and persisted as a cause of invasive disease during 2000–2009. A plausible explanation for this clonal emergence is the maintenance of virulence after capsular switch and a lack of immunity in a portion of the population in RJ. One of the clones associated with the serogroup B epidemic, B:3–1:P1.19.15:F5–1:ST-33(32CC), against which mass vaccination was directed, resulted in only 2 serogroup C cases.

Capsular switching represents a mechanism by which meningococci escape protective immunity directed at specific capsular polysaccharides (*4*). The emergence and expansion of the serogroup W135 Hajj clone in the setting of vaccination against serogroups A and C illustrate this phenomenon (*11*). However, capsular switching does not always lead to clonal emergence. For example, the serogroup C clone that resulted from capsular switching within the serogroup B epidemic in Oregon has caused relatively few cases (*5*).

We report a substantial number of cases of meningococcal disease and case clusters during 2000–2009 that were associated with ST32/ET-5 serogroup C clones that had emerged in Brazil during 1993–1998. These strains might retain the attributes of the lineages of the ST32 complex B clones, including a propensity to cause outbreaks. Additionally, some capsular variants may have a selective advantage and might, at least in part, replace original strains circulating in the population (*12*). In studies of *N. meningitidis* ST11CC strains bearing serogroups C or W135 in Brazil (*13**,**14*) and serogroups C and B in Spain (*15*), a pattern of preserved hyperinvasiveness in the emerged W135 and B strains was also found. The ability of *N. meningitidis* to cause substantial disease after capsular switching highlights the need for surveillance of circulating meningococcal strains to delineate optimal disease control policies.
